# Insights into Structure and Biological Activity of Copper(II) and Zinc(II) Complexes with Triazolopyrimidine Ligands

**DOI:** 10.3390/molecules27030765

**Published:** 2022-01-24

**Authors:** Aura Argăseală, Cătălin Maxim, Mihaela Badea, Larisa Ioniță, Mariana Carmen Chifiriuc, Arpad Mihai Rostas, Mihaela Bacalum, Mina Răileanu, Lavinia L. Ruţă, Ileana C. Farcaşanu, Emilia Elena Iorgulescu, Rodica Olar

**Affiliations:** 1 Department of Inorganic Chemistry, Faculty of Chemistry, University of Bucharest, 90–92 Panduri Str., 050663 Bucharest, Romania; aura.argaseala@s.unibuc.ro (A.A.); catalin.maxim@chimie.unibuc.ro (C.M.); mihaela.badea@chimie.unibuc.ro (M.B.); 2 Doping Control Laboratory, 37–39 Basarabia Blvd., 022103 Bucharest, Romania; l.ionita@lcd.gov.ro; 3 Department of Microbiology, Faculty of Biology, University of Bucharest, 1–3 Aleea Portocalelor Str., 060101 Bucharest, Romania; carmen.chifiriuc@bio.unibuc.ro; 4 Academy of Romanian Scientists, 010071 Bucharest, Romania; 5 Biological Sciences Division, The Romanian Academy, 010071 Bucharest, Romania; 6 Laboratory of Atomic Structures and Defects in Advanced Materials, National Institute of Materials Physics, 405A Atomiştilor Str., 077125 Măgurele-Ilfov, Romania; arpad.rostas@infim.ro; 7 National Institute for Research and Development of Isotopic and Molecular Technologies, 67–103 Donat, 400293 Cluj-Napoca, Romania; 8 Department of Life and Environmental Physics, Horia Hulubei National Institute for Physics and Nuclear Engineering, 30 Reactorului Str., 077125 Măgurele-Ilfov, Romania; bmihaela@nipne.ro (M.B.); mina.raileanu@nipne.ro (M.R.); 9 Department of Electricity, Solid State and Biophysics, Faculty of Physics, University of Bucharest, 405A Atomiştilor Str., 077125 Măgurele-Ilfov, Romania; 10 Department of Organic Chemistry, Biochemistry and Catalysis, Faculty of Chemistry, University of Bucharest, 90–92 Panduri Str., 050663 Bucharest, Romania; lavinia.ruta@chimie.unibuc.ro (L.L.R.); ileana.farcasanu@chimie.unibuc.ro (I.C.F.); 11 Department of Analytical Chemistry, Faculty of Chemistry, University of Bucharest, 90–92 Panduri Str., 050663 Bucharest, Romania

**Keywords:** complex, 1,2,4-triazolo[1,5-*a*]pyrimidine, cytotoxicity, biofilm, metallonuclease activity

## Abstract

In an attempt to increase the biological activity of the 1,2,4-triazolo[1,5-*a*]pyrimidine scaffold through complexation with essential metal ions, the complexes *trans*-[Cu(mptp)_2_Cl_2_] (**1**), [Zn(mptp)Cl_2_(DMSO)] (**2**) (mptp: 5-methyl-7-phenyl-1,2,4-triazolo[1,5-*a*]pyrimidine), [Cu_2_(dmtp)_4_Cl_4_]·2H_2_O (**3**) and [Zn(dmtp)_2_Cl_2_] (**4**) (dmtp: 5,7-dimethyl-1,2,4-triazolo[1,5-*a*]pyrimidine), were synthesized and characterized as new antiproliferative and antimicrobial species. Both complexes (**1**) and (**2**) crystallize in the *P*2_1_/n monoclinic space group, with the tetrahedral surroundings generating a square-planar stereochemistry in the Cu(II) complex and a tetrahedral stereochemistry in the Zn(II) species. The mononuclear units are interconnected in a supramolecular network through π–π interactions between the pyrimidine moiety and the phenyl ring in (**1**) while supramolecular chains resulting from C-H∙∙∙π interactions were observed in (**2**). All complexes exhibit an antiproliferative effect against B16 tumor cells and improved antibacterial and antifungal activities compared to the free ligands. Complex (**3**) displays the best antimicrobial activity against all four tested strains, both in the planktonic and biofilm-embedded states, which can be correlated to its stronger DNA-binding and nuclease-activity traits.

## 1. Introduction

The problems related to drug resistance in cancer and microbial infections are complex and lead to thousands of deaths each year. These adverse outcomes originate from the limitations associated with organic antibiotics [1,2] and cytostatics [3], including inorganic drugs like cisplatin. Cisplatin and its analogs are currently used to treat cancer, but they often lead either to severe side effects or acquired resistance [4]. As a result, the complexes bearing essential elements represent an alternative strategy to overcome these shortcomings, considering the human body’s ability to manage their excess, either by storage or by excretion [5].

Among these species, complexes with Cu(II) and Zn(II) proved to be valuable in developing bioactive compounds with potential applications in several biomedical fields due to their antimicrobial [6,7,8], antitumor [8,9,10,11,12,13,14,15,16], antiparasitic [17], or anti-inflammatory [8] activities. These ions are usually selected for this purpose based on their low systemic toxicity, borderline acid character, and stereochemical versatility, allowing their interaction with a wide range of biomolecules in different biological systems.

Studies concerning the complexes with such ions evidenced differences concerning their mechanisms of action. Thus, while Cu(II) manifests both redox [18] and hydrolytic [19] abilities in a biological environment, Zn(II) is primarily involved in promoting the hydrolytic reactions as a result of its strong Lewis-acid character [5]. Moreover, the interaction with biomolecules can be assisted by ligands that can control both the complex stability and their interaction with aromatic components of DNA and proteins through intercalation and/or weak interactions such as dipole–dipole, electrostatic, hydrogen bonds, or π–π stacking.

From this point of view, a valuable scaffold is a triazolopyrimidine-fused ring selected for biologically-active-complex development considering its resemblance to the purine bases [20] and proven ability to intercalate into DNA strands [21]. Furthermore, several complexes of Cu(II) and Zn(II) with triazolopyrimidine derivatives (tpds) have been already proven to be valuable antiparasitic, antimicrobial or antitumor species [17,20].

Thus, high in vitro activity against the extracellular forms of both *Leishmania* spp. (promastigote) and *Trypanosoma cruzi* (epimastigote), coupled with a low toxicity against macrophage host cells was evidenced for M(tpds)n^2+^ units [M: Cu, Zn, tpds: 5,7-dimethyl-1,2,4-triazolo[1,5-*a*]pyrimidine (dmtp) [22,23,24], 7-amino-1,2,4-triazolo [1,5-*a*]pyrimidine (7atp) [25], 5-phenyl-1,2,4-triazolo[1,5-a]pyrimidi-7(4H)-one (HftpO)] [26] or 7-amino-5-methyl-1,2,4-triazolo[1,5-*a*]pyrimidine (7amtp) [27] and *n* = 2,4), which are usually associated with chloride, perchlorate or nitrate counter-ions. Moreover, a series of complexes of the two metallic ions with mixed ligands, tpds and 1,10-phenanthroline (phen) [28], ethylenediamine [28] or 2,2’-bipyrimidine [29], proved to be active against the parasites responsible for leishmaniasis and Chagas disease.

Besides the antiparasitic activity, a glucose-lowering potential was recently reported for different hydrated species containing the moiety Zn(7amtp)_2_X_2_ (X: Cl, NO_3_, SO_4_). All of these complexes present a higher selectivity index than the above-described species, and mice experiments have confirmed their ability to reduce glucose levels after oral administration [27].

A broad spectrum of antimicrobial activity against both planktonic and biofilm-embedded strains, including methicillin-resistant *Staphylococcus aureus* (MRSA), was demonstrated by a series of complexes of 5-methyl-7-phenyl-1,2,4-triazolo[1,5-a]pyrimidine (pmtp) with the M(pmtp)X_2_ moiety (M: Cu, Zn; X: Cl [30], CH_3_COO [31] and ClO_4_ [32]). Furthermore, the Cu(II) species with mixed ligands, pmtp or dmtp and 2,2’-bipyridine (bpy) or 1,10-phenanthroline, showed enhanced activity in comparison with both ligands against several planktonic and biofilm-embedded bacterial strains [21,33].

The acetate and perchlorate species of Cu(II) with pmtp exhibited moderate antiproliferative activity against the human colon adenocarcinoma cell line (HT 29) [31,32]. In contrast, Cu(II) complexes with pmtp/dmtp and bpy/phen as auxiliary ligands exhibited excellent activity against B16 melanoma cells, accompanied by a low toxicity in eukaryotic cells [21,33].

**Figure 1:**
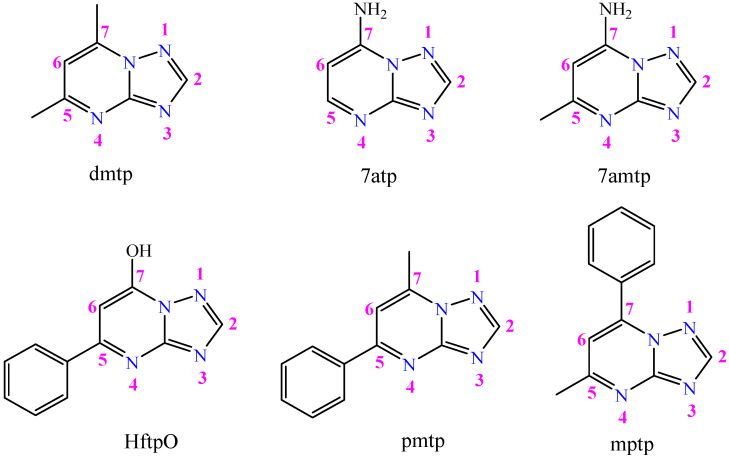
The structure for 1,2,4-triazolo[1,5-a]pyrimidine derivatives described in the paper together with IUPAC ring-numbering system.

The structure for 1,2,4-triazolo[1,5-a]pyrimidine derivatives described in the paper is presented in Figure 1.

The resistance to organic drugs observed in both pathogenic microorganisms and tumor cells as well as infections associated with microbial biofilms represent one of the main research challenges in developing new, efficient and non-toxic species. One of the most promising molecular targets is represented by DNA replication, and, in this context, high antimicrobial and antitumor activity can be exhibited by species able to interact with the DNA, which could inhibit the multiplication for both the pathogenic microorganism (in planktonic and biofilm growth state) and the neoplastic process, which is characterized by an uncontrolled cellular proliferation. A strong interaction at the DNA level can be achieved by the common action of the metal ion and the ligand.

In this context, the purpose of this paper was to combine the ability of Cu(II) and Zn(II) to coordinate DNA and to generate reactive oxygen species (ROS) with the capacity of tpds to establish π–π stacking interactions with aromatic components of DNA. As a result, we have obtained and characterized a new series of chloride complexes of Cu(II) and Zn(II) with mptp and dmtp, developed as species with antiproliferative activity against B16 melanoma cells and antibiofilm activity against some pathogenic bacteria and *C. albicans* fungal strains. The chloride was selected as a counter-ion based on both its coordinative ability and its presence in normal biological fluids. These properties could assure an enhanced lipophilicity for neutral complexes and a low toxicity upon metabolization.

The complexes were characterized by single-crystal X-ray analyses and by different spectroscopic methods. Their stability during biological assays was proved by NMR and EPR experiments. The complexes’ interaction with relevant biological systems, as well as their possible mechanisms of actions, such as their interaction with DNA and ROS release, were also investigated.

## 2. Results and Discussions

**Scheme 1:**
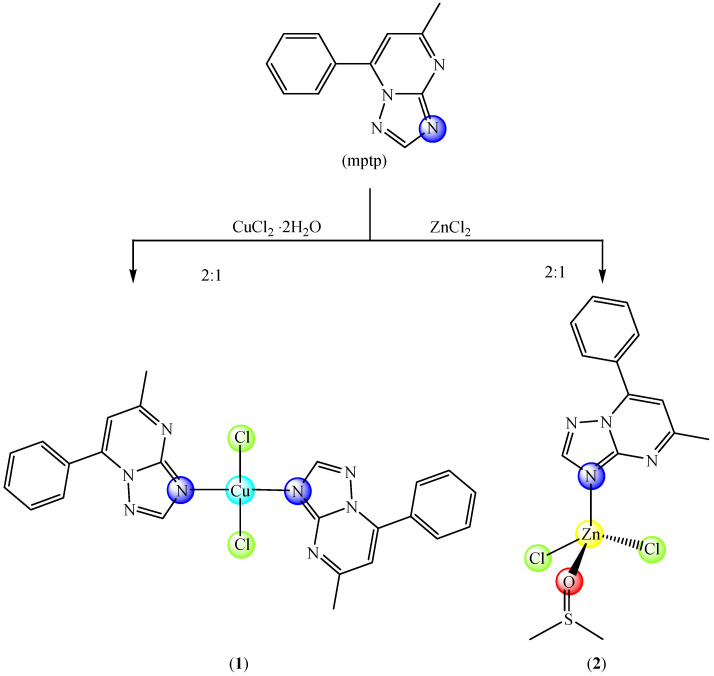
The synthesis route of *trans*-[Cu(mptp)_2_Cl_2_] (**1**) and [Zn(mptp)Cl_2_(DMSO)] (**2**).

New complexes of copper(II) and zinc(II) with 5-methyl-7-phenyl-1,2,4-triazolo[1,5-*a*]pyrimidine (mptp) were synthesized by the ligand direct reaction with metal chlorides in an ethanol–dimethylsulfoxide (DMSO) mixture, as presented in Scheme 1. Although the stoichiometric ratio between the salts and organic ligands was 1:2, in the case of Zn(II), the compound corresponding to the 1:1 ratio was obtained.

**Scheme 2:**
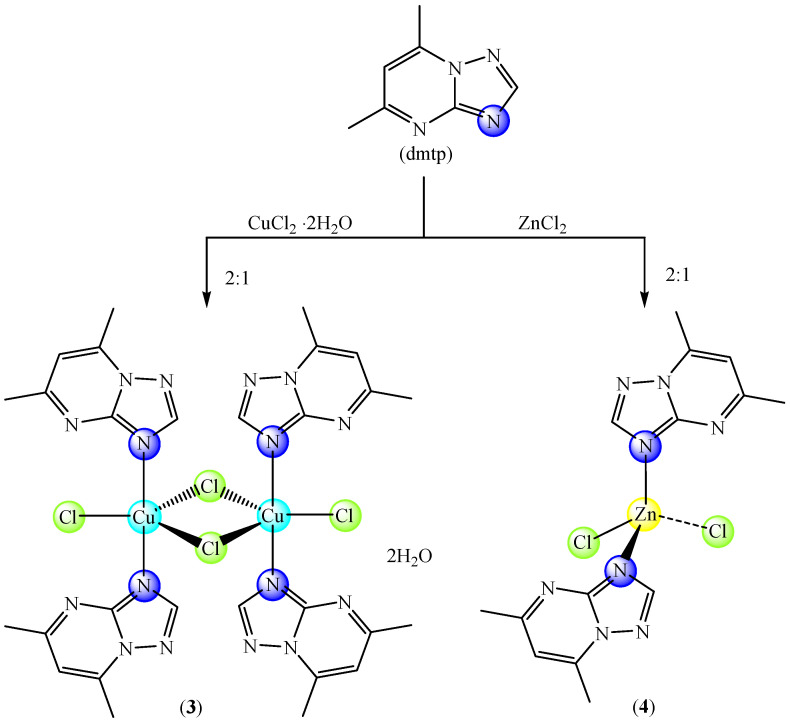
The synthesis route of [Cu_2_(dmtp)_4_Cl_4_]·2H_2_O (**3**) and [Zn(dmtp)_2_Cl_2_] (**4**).

The corresponding species with 5,7-dimethyl-1,2,4-triazolo[1,5-a]pyrimidine (dmtp) (Scheme 2) were synthesized according to a slightly modified, previously published method [22,34] in order to investigate the substituents’ influence on biological activity. The new compounds were fully characterized by single-crystal X-ray diffraction. Additional data consisting of EPR, FTIR, and UV-Vis spectra and redox behavior were also collected and are discussed in the following sections. Although the complexes (**3**) and (**4**) have been already characterized from the crystallographic and spectroscopic points of view [21,33], their EPR, UV-Vis, NMR, and cyclic voltammetry data are presented here for the first time together with their antimicrobial and antiproliferative activity.

### 2.1. Description of the Crystal Structure of Complexes

**Figure 2:**
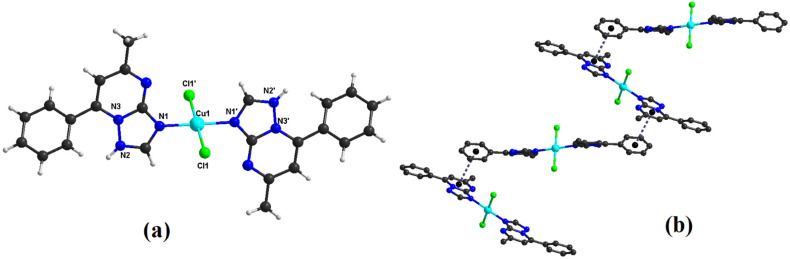
The crystal structure for (**1**) with atoms labeled (**a**) and the supramolecular chains in compound (**1**) (**b**).

Slow evaporation of the ethanolic solution of *trans*-[Cu(mptp)_2_Cl_2_] (**1**) led to a mononuclear coordination complex (Figure 2a). Compound (**1**) crystallized in the *P*2_1_/n monoclinic space group, with cell parameters and structure-refinement details given in Table 1.

The asymmetric units consist of one crystallographically independent Cu(II) ion lying on the inversion center, one 5-methyl-7-phenyl-1,2,4-triazolo[1,5-a]pyrimidine molecule (mptp), and one chlorine ion. The Cu1 atom is tetra-coordinated, showing a slightly distorted square-planar geometry as indicated by angles of about 90° around the metal ion (Supplementary Table S1). The plane of the metal ion is occupied by two nitrogen atoms from the mptp ligand [Cu1-N1 = 1.986(2) Å], while the chlorine ligands fill the *trans* positions [Cu1-Cl1 = 2.2583(8) Å] (Table 2).

Regarding the crystal-packing analysis, one can observe that the mononuclear units are interconnected through aromatic π–π interactions occurring between the triazolopyrimidine ligands coordinated to the copper ions and the phenyl ring. Supramolecular chains are thus formed (Figure 2b).

**Figure 3:**
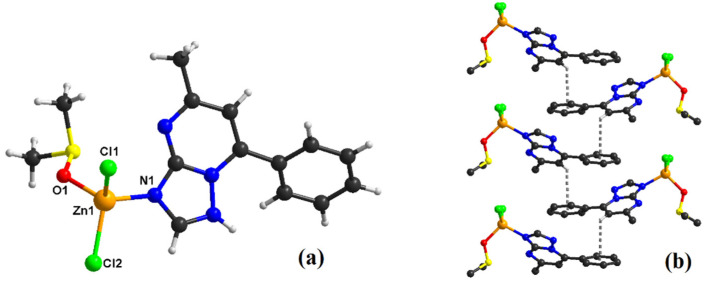
Crystal structure of (**2**): view of the asymmetric unit (**a**) and supramolecular chains resulting from C-H∙∙∙π interactions (**b**).

The crystal structure of compound (**2**) (Figure 3a) consists of mononuclear [Zn(mptp)Cl_2_(DMSO)] species, in which the metal ion is connected to two chlorine units, one triazolo-derivative ligand, and one DMSO molecule. Each zinc(II) ion is tetra-coordinated, having a distorted tetrahedral geometry that generates the corresponding angles of 107° around the metal ion (Supplementary Table S1). Therefore, two positions are occupied by two chlorine atoms (Zn1–Cl1 = 2.2133(8) Å; Zn1–Cl2 = 2.2234(9) Å), one imine-type nitrogen atom (Zn1–N1 = 2.033(2) Å), and one oxygen atom from the DMSO ligand (Zn1–O1 = 1.9921(18) Å), respectively.

The crystal packing of compound (**2**) is governed by π–π stacking interactions (Figure 3b). Thus, the mononuclear assemblies communicate through π–π stacking interactions (edge to face) between the phenyl ring and the hydrogen atom from an adjacent N-heterocycle. The self-assembly of these non-covalent interactions constructs supramolecular chains running along the *b* axis.

A comparison of the M-N_tpds_ bond lengths (Table 3) evidenced slightly shorter bonds for Cu(II) complexes (**1**) and (**3**) in comparison with the corresponding Zn(II) species, and a similar trend can be noticed for the mptp compounds in comparison with those bearing dmtp as a ligand. Instead, for unidentate chloride ligands, the M-Cl bond lengths are shorter for the Zn(II) species, while the longest bond is observed in the bridge ligands Cu-Cl-Cu in complex (**3**).

### 2.2. Physico-Chemical Characterization of Complexes

#### 2.2.1. FT-IR Spectra

The complexes’ spectra reveal the presence of two strong bands in the 1540–1635 cm^−1^ range, assigned to the stretching vibrations of the triazolopyrimidine moiety (νtp) and pyrimidine ring (νpym), respectively. These bands are slightly shifted to higher or lower wavenumbers for all complexes than for the free ligands, similar to other complexes with tpds coordinated through the N^3^ atom [21,22,23,24,25,26,27,28,29,30,31,32,33]. The additional low-intensity bands in the 455–480 cm^−1^ range can be assigned to the ν(M-N) stretching vibration [35]. The other bands noticed at 1026 and 722 cm^−1^ in the complex (**2**) spectrum are assigned to υ (S=O) and υ(C-S) vibrations for coordinated DMSO [35].

#### 2.2.2. UV-Vis Spectra

A comparison of the spectra obtained for complexes (**1**) and (**2**) with those of mptp reveals that the bands assigned to the intraligand π→π* transition appear at 380 nm in the mptp spectrum and are shifted to a lower wavelength in the complexes’ spectra as a result of coordination. In the visible region of the diffuse-reflectance spectra of complex (**1**), an unsymmetrical absorption band split into three components with absorption maxima at 560, 610, and 660 nm can be noticed (Supplementary Figure S1); this pattern is characteristic to the Cu(II) ion in a distorted square-planar stereochemistry [36]. The supplementary band at 350 nm is assigned to ligand to metal charge transfer (LMCT) transition.

For dmtp, the π→π* transition band appears at 300 nm with a shoulder at 490 nm and is similarly shifted in the complexes’ spectra (Supplementary Figure S2). The spectrum of complex (**3**) displays the supplementary bands assigned to the d–d transitions at 750 and 910 nm and the transition accounting for LMCT at 345 nm, which is an aspect characteristic of the trigonal pyramidal environment of Cu(II) [36].

#### 2.2.3. EPR Spectroscopy

##### Solid State EPR Spectroscopy

**Figure 4:**
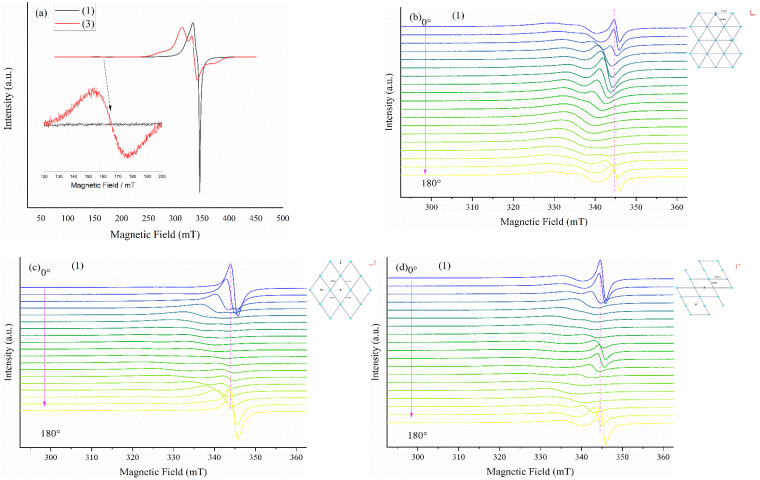
Powder EPR spectra of the complexes (**1**) and (**3**) at room temperature (**a**), EPR spectra of a single crystal of complex (**1**) with B_0_ perpendicular to the *ca* (**b**), *ab* (**c**), and *bc* (**d**) planes, with rotation along the *c*, *a*, and *b* axes.

Figure 4a shows the powder EPR spectra of the two complexes (**1**) and (**3**). Both spectra present axial g-values with g‖ = 2.12246 and g_⊥_ = 2.0438 for complex (**1**) and g‖ = 2.1295 and g_⊥_ = 2.0696 for complex (**3**). Because of the strong spin–spin exchange interaction, no hyperfine coupling is observable. The formation of dimers in complex (**3**) is evidenced by the EPR signal at half field, which is much lower in intensity and depicted in the inset of Figure 4a.

Figure 4b–d show the EPR spectra of a single complex (**1**) crystal with B0 perpendicular to the ca, ab, and bc planes, respectively, with rotation along the c, a, and b axes, respectively. The spectra presented in Figure 4b,c show a strong exchange interaction evidenced by a sharp EPR signal for the single-crystal oriented perpendicular to the z-axis and a weak interaction for the 90° rotation along the axis mentioned above. For the orientation presented in Figure 4d, a strong exchange interaction is also observable for the 90° position. These findings are in excellent agreement with the crystal structure shown in the inset of each figure.

##### Solution EPR Spectroscopy

**Figure 5:**
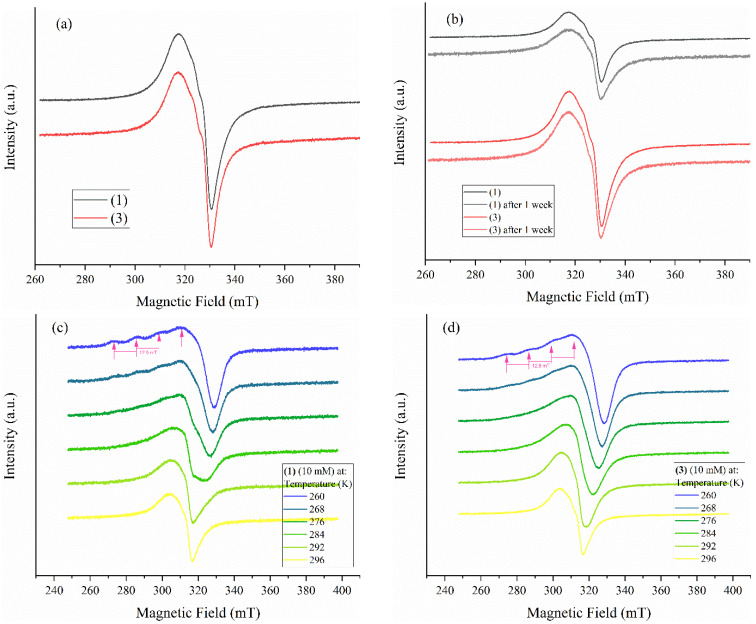
EPR spectra of complexes (**1**) and (**3**) in 10 mM DMSO solution: freshly prepared (**a**), after one week (**b**), and at temperatures ranging from 260 to 296 K for (**1**) (**c**) and (**3**) (**d**).

The biological assays of the complexes were carried out in a DMSO solution and their stability was monitored over one week through the modifications in their EPR spectra. A 10 mM solution was used for this experiment since the EPR spectra of lower concentrations showed no changes (Supplementary Figure S3) in the line width or any additional hyperfine lines. Still, the spectra had a lower signal-to-noise ratio, making the accumulation time 100 times longer. Figure 5a shows the EPR spectra of the Cu(II) complexes in the DMSO solution having almost identical line shapes. This behavior is predictable even if the structures of the two complexes is different, as described in the previous sections. Hence, in solution, the Cu-Cl-Cu bonds of complex (**3**) break, and the spin–spin interaction observed earlier disappears, leaving the copper center of complex (**3**) in the same conditions as that of complex (**1**). This aspect is in agreement with the complexes’ structures described above.

The stability of complexes (**1**) and (**3**) in a DMSO solution is presented in Figure 5b. The line shape and intensity of the EPR spectra present no changes, showing excellent stability of the Cu(II) complexes even after this long period.

The compounds with a 10 mM concentration were also measured at temperatures ranging from 260 to 296 K (Figure 4c,d). The low-temperature EPR spectra (at 260 K) present the same g-values as presented in the previous section. Because of the low concentration, the hyperfine coupling is revealed with A_‖_ = 12.8 mT, indicating that the structure of the complexes is maintained in the DMSO solution. The temperature sweep suggests that the mobility of the two complexes is slightly different, as evidenced by the different line shapes of the EPR spectra recorded at temperatures between 260 and 296 K. The ligand of complex (**1**) is somewhat larger than that of complex (**3**), which directly influences its mobility in solution, and the EPR spectra become isotropic only at 296 K compared to complex (**3**), where the isotropy occurs at 284 K. This observation is in excellent agreement with the proposed structures of the molecules.

#### 2.2.4. NMR Spectroscopy

The ^1^H NMR spectrum of (**2**) exhibits singlets at 2.54 and 2.68 ppm assigned to the CH_3_ groups of both the DMSO and pmpt ligands. On the other hand, other signals at 8.61 and 7.54 ppm also appear as singlets and are characteristic of the proton of the triazole and pyrimidine groups, respectively [37]. The five protons of the phenyl moiety generate a doublet at 8.15 ppm and a multiplet at 7.64 ppm (Supplementary Figure S4).

The ^13^C NMR spectrum of complex (**2**) displays signals at 16.94 (CH_3_), 24.62 (DMSO-CH_3_), 110.40, 146.17, 165.23 (pyrimidine ring), 128.65, 129.09, 129.50, 129.67, 131.53 (phenyl ring), and 155.28 ppm (triazole ring), respectively (Supplementary Figure S5).

It is worth mentioning that the DMSO presence as a ligand is responsible for the signals at 2.54 ppm in the ^1^H NMR spectrum and at 24.62 ppm in the ^13^C NMR spectrum. Moreover, the spectrum of this complex is not modified after 72 h (Supplementary Figure S4). The same trend was observed after 72 h for complex (**4**) (Supplementary Figure S6), indicating the complexes’ stability in DMSO during the biological experiments.

The signals characteristic of the protons of triazole and pyrimidine rings appear at 7.18 and 8.56 ppm, respectively. By comparison, the signals of the two methyl groups are observed at 2.58 and 2.73 ppm in the ^1^H NMR spectrum of compound (**4**) (Supplementary Figure S6). The carbon atoms from this complex are confirmed by the signals at 16.45, 24.47 (CH_3_), 110.90, 147.04, 164.54 (pyrimidine ring), and 155.06 ppm (triazole ring), respectively (Supplementary Figure S7).

#### 2.2.5. Voltammetric Studies

The electrochemical properties of the complexes were studied by cyclic voltammetry, considering that the redox potential influences their interaction with another redox-active species in biological systems.

**Figure 6:**
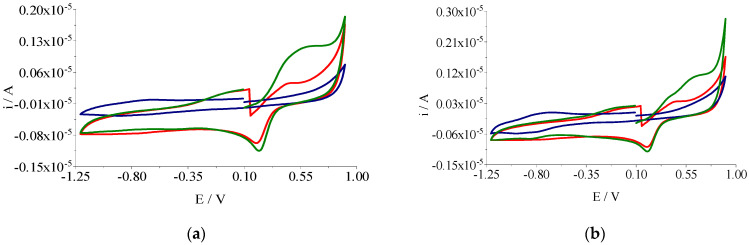
Cyclic voltammograms of copper complex (**1**) (**a**) and complex (**3**) (**b**), cyclic voltammograms of [Cu(DMSO)_6_]Cl_2_ (red line), ligand (blue line), complex _(_green line), 1 mM in DMSO, (0.1 M Bu_4_NClO_4_; scan rate: 0.050 V/s, working electrode, platinum disk, reference electrode, Ag/AgCl (0.1 M Bu_4_NClO_4_ in DMSO).

The study of the electrochemical behavior of the Cu(II) complexes (**1**) and (**3**) was carried out to investigate the oxidation-reduction process, in the range of +0.10 → +0.90 V → −1.20 V (Figure 6). In the positive range, the oxidation processes of Cu(II)/Cu(III) can be observed at E_pa1_ = +0.481 V for complex (**1**) and E_pa1_ = +0.583 V for complex (**3**), with values shifted to a positive range compared to those obtained for the [Cu(DMSO)_6_]Cl_2_ species, leading to higher values of E_1/2_ proper for the hydrogen peroxide reduction. Cyclic scanning between +0.90 and −1.20 V shows a large reduction counter-peak at E_pc1_ = +0.200 V for all copper compounds (Table 4). For complex (**3**), a reduction wave at E_pc2_ ~ −0.800 V is also observed, which can be assigned to the reduction of both the ligand and Cu(II)/Cu(I).

**Figure 7:**
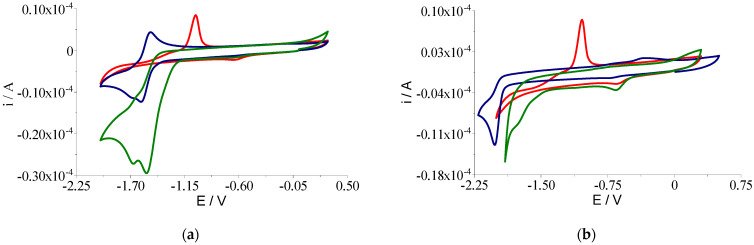
Cyclic voltammograms of zinc complex (**2**) (**a**) and complex (**4**) (**b**), cyclic voltammograms of [Zn(DMSO)_4_]Cl_2_ (red line), ligand (blue line), complex (green line), 1 mM in DMSO (scan rate: 0.100 V/s, working electrode: glassy-carbon disk, reference electrode: Ag/AgCl (0.1 M Bu_4_NClO_4_ in DMSO)).

Cyclic voltammetric scans of zinc complexes, [Zn(DMSO)_4_]Cl_2_ and ligands (mptp and dmtp) at a concentration of 1 mM in DMSO using tetrabutylammonium perchlorate (Bu_4_NClO_4_) 0.1 M as the supporting electrolyte, with a sweep rate of 0.1 V s^−1^ and a glassy-carbon-disk electrode were obtained and are presented in Figure 7.

In the case of [Zn(DMSO)_4_]Cl_2_ in DMSO solution, a two-electron reduction (E_pc1_ = −0.635 V) can be observed such that the back scan exhibits the typical ’anodic stripping’ of the electrodeposited Zn(0) at E_pa1_ = −1.037 V. The cyclic voltammograms recorded for the zinc-complex solutions (**2**) and (**4**) exhibit an irreversible reduction wave (E_pc1_ = 0.650 V) followed by further ligand-centered irreversible processes (E_pc2_ = 1.531 V and E_pc2_ = 1.742 V), with mptp and dmtp being the electrochemically active ligands. Comparing the values of the peak potential corresponding to the Zn(II) reduction, it is evident that complexation does not render the reduction process more difficult (Table 5).

**Figure 8:**
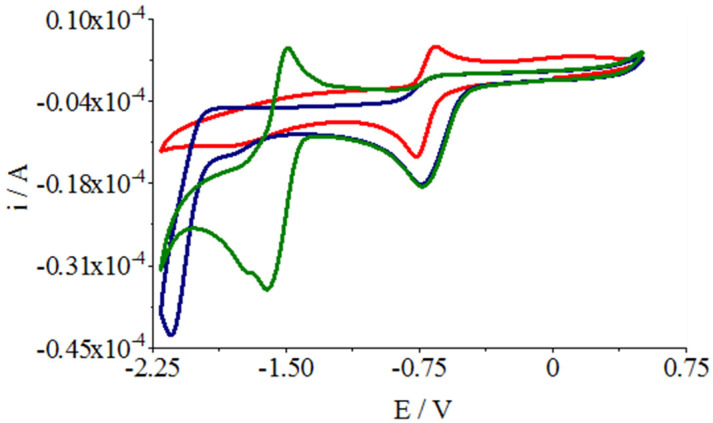
Cyclic voltammograms of the electrolyte solution, 0.1 M Bu_4_NClO_4_ in DMSO without de-aeration with argon (red line), complex (**2**) (green line) and complex (**4**) (blue line), complexes’ concentration 1 mM in DMSO without de-aeration (scan rate: 0.100 V/s, working electrode: glassy-carbon disk: reference electrode, Ag/AgCl (0.1 M Bu_4_NClO_4_ in DMSO)).

The zinc(II) complexes’ effect on the superoxide disproportionation was evaluated by cyclic voltammetry in DMSO without de-aeration with argon. In the absence of the zinc complexes, the wave for the quasi-reversible reduction of the O_2_ to O_2_^−^ (Epc = −0.765 V and Epa = −0.664 V, red cyclic voltammogram in Figure 8) was observed. When the zinc(II) complexes were added to the solution, the wave corresponding to the O_2_^−^ oxidation decreased by approximately tenfold in intensity. This implies that the zinc complexes quench the superoxide in solution (blue and green cyclic voltammograms in Figure 8) [38].

### 2.3. Complexes Interactions with Cells and Biological Species

#### 2.3.1. Antiproliferative Activity

The organic cytostatics used for melanoma chemotherapy generate severe side effects, including an increased risk of infection as well as resistance development. Hence, in a continuous search for new antitumor drugs with reduced side effects and lower toxicity, the Cu(II) complexes are preferred, considering their reduced systemic toxicity [18]. Thus, several Cu(II) species were developed as non-toxic compounds for healthy tissue but with high activity in melanoma cells [33,39,40].

**Figure 9:**
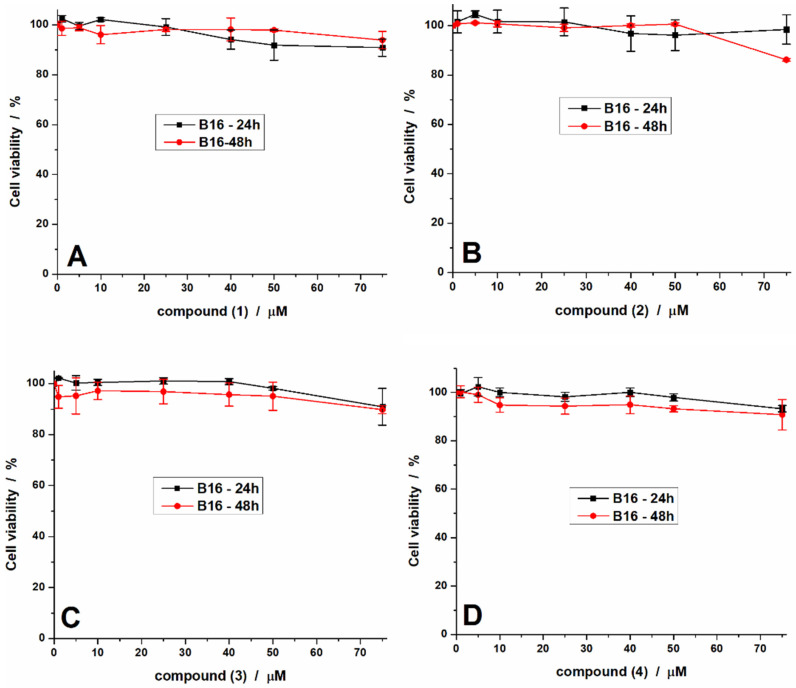
The cytotoxic effect of the compounds (**1**)—(**A**), (**2**)—(**B**), (**3**)—(**C**) and (**4**)—(**D**) on B16 cells evaluated after 24 and 48 h (each value represents the mean ± SD).

The compounds reported in this study were tested against B16 cells for 24 and 48 h, and data are presented in Figure 9. Similar to the mptp and dmtp precursors, the results show the reduced toxicity of all the complexes against the cells at concentrations below 25 µM. However, the viability of the cells decreased to about 80% for a concentration of 75 µM, indicating that the compounds reduce cell viability.

**Figure 10:**
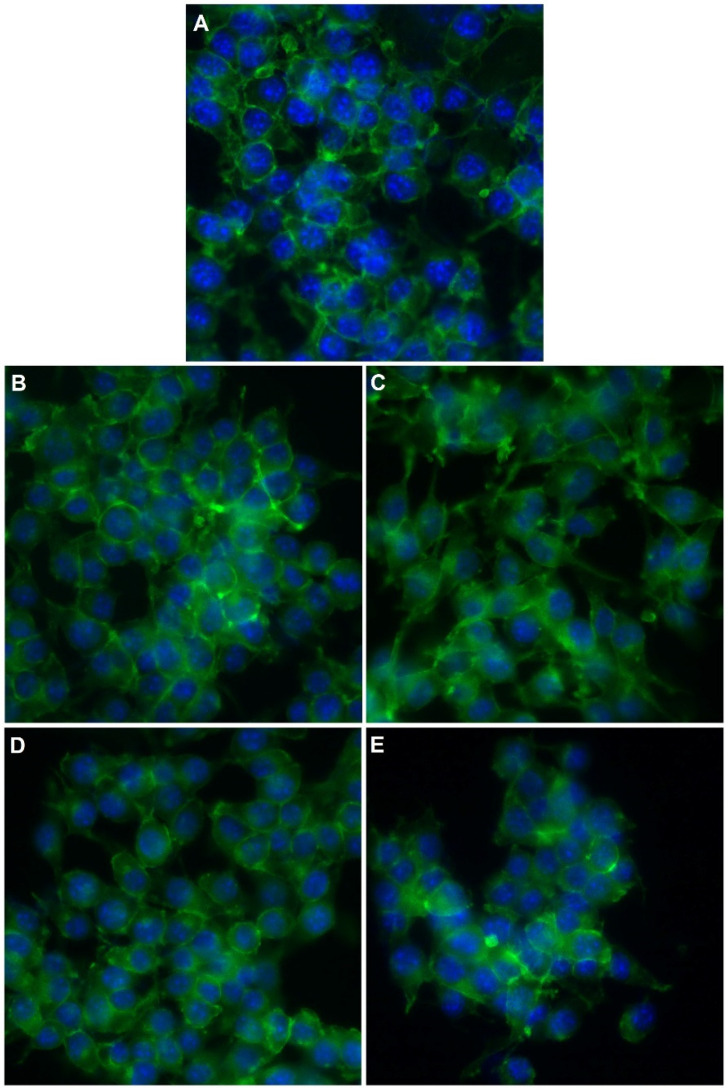

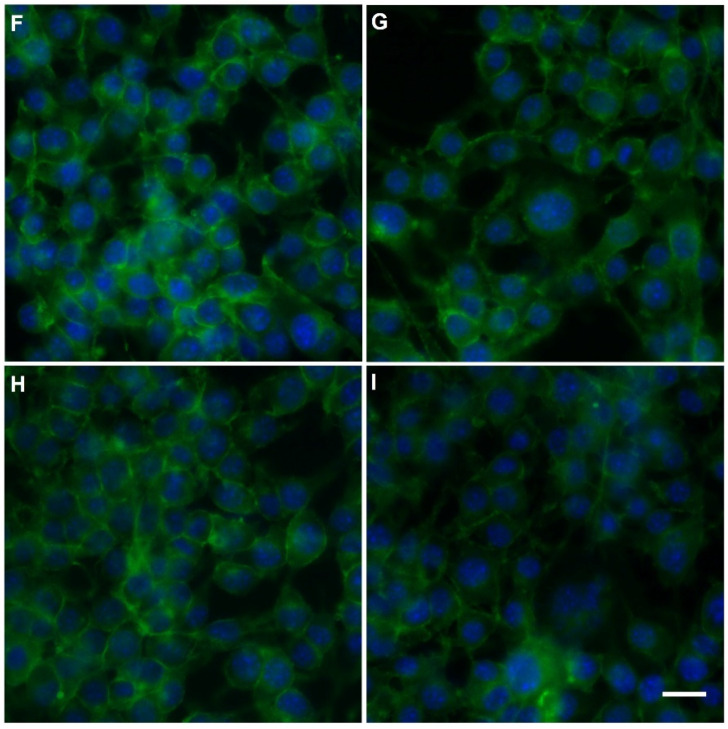
Cell morphology of B16 cells: control cells (**A**), treated with 25 µM (**B**) and 75 µM (**C**) compound (**1**), treated with 25 µM (**D**) and 75 µM (**E**) compound (**2**), treated with 25 µM (**F**) and 75 µM (**G**) compound (**3**) and treated with 25 µM (**H**) and 75 µM (**I**) compound (**4**) for 24 h. Cell nuclei are stained with Hoechst 33342 (blue), and actin filaments are stained with Phalloidin-FITC (green). The scale bar is 20 μm and the same for all images.

In order to determine how the compounds affect the cell morphology, the nucleus and cytoskeleton of the cells were marked for those treated with concentrations of 25 and 75 µM. The results presented in Figure 10 show that the morphology of the cells treated with a 25 µM solution of the compounds is not changed, and a similar number of cells appears. The cells treated with 75 µM also show no significant changes. However, the number of cells is reduced, indicating that the treatments kill the cells.

Considering the results reported in this paper and the previous studies [21,33], we can conclude that the complexes exhibit an antiproliferative effect against the B16 tumor cells at concentrations higher than 75 µM. However, these concentrations are high in comparison with the half-maximal inhibitory concentration (IC_50_) values recorded at 48 h for other species with tpds ligands, such as [Cu(N-N)_2_(pmtp)](ClO_4_)_2_ (IC_50_ 20.00 and 4.00 μM) [21] and [Cu(N-N)(dmtp)_2_(OH_2_)](ClO_4_)_2_·dmtp (N-N: 2,2′-bipyridine or 1,10-phenanthroline) (IC_50_ 4.42 and 4.58 μM) [33]. These data suggest the necessity of a heterocyclic-derivative presence as an auxiliary ligand in complexes with tpds in order to achieve a high antiproliferative activity against B16 tumor cells.

#### 2.3.2. Microbiological Activity

Opportunistic infections are often a complication of oncological treatment due to the immunosuppression and intestinal dysbiosis induced by the antitumor agents [41,42,43]. Therefore, if the antitumor species could also exhibit an antimicrobial activity directed towards opportunistic bacterial and fungal pathogens, this could represent an advantage and would be associated with a lower risk of side effects and complications.

The antimicrobial activity of the complexes was evaluated against the Gram-negative (*Klebsiella pneumoniae* ATCC 134202, *Pseudomonas aeruginosa* ATCC 27853) and Gram-positive (*Bacillus subtilis* ATCC 6633) reference bacterial strains, as well as the *Candida albicans* 22 fungal strain. The antimicrobial activity of the ligands, metal chlorides, and complexes was evaluated against planktonic and biofilm-embedded cells using quantitative assays, allowing us to establish the minimum inhibitory (MIC) and minimum biofilm eradication (MBEC) concentrations, respectively.

In the case of complexes (**1**) and (**2**), bearing the pmpt as ligand, they showed a significantly improved antibacterial activity as compared to that of the free ligand and metal salts, taking into account the much lower MIC values obtained for (**1**) and (**2**) (Table 6). Both complexes showed excellent antibacterial activity. Still, the lowest MIC values were obtained against *K. pneumoniae,* which is known as one of the most frequent etiological agents of opportunistic infections, such as bacteremia, in cancer patients, with worse prognoses when resistant strains are involved [44].

In the case of *C. albicans* 22, only complex (**2**) exhibited antifungal activity.

Complexes (**3**) and (**4**), bearing dmpt as ligand, also exhibited much improved antibacterial and antifungal activity compared to the ligand. Complex (**3**) showed the best antimicrobial activity, as revealed by the shallow MIC values obtained for all four tested strains.

The *K. pneumoniae* strain proved to be the most susceptible to all four complexes, their efficiency decreasing in the following order (**4**) > (**3**) > (**2**) > (**1**). However, when all four tested strains are considered, the efficiency order changes as follows (**3**) > (**2**) > (**1**) > (**4**).

It is well known that both in the external environment and clinical infections, microorganisms are often adherent to different surfaces and interfaces, forming sessile, multicellular associations known as biofilms. Many mechanisms protect these communities from various limiting factors, including antimicrobial agents [45]. Therefore, in general, the anti-biofilm concentrations are much higher than those required to eradicate free microbial cells. However, in the case of our complexes, the average values were very similar, end even lower than the corresponding MIC values for complexes (**1**), (**2**), and (**3**).

Similar to the MIC assays, the obtained complexes showed a much more improved antibacterial and antifungal activity as compared to that of the ligand. The most susceptible biofilm was that formed by *K. pneumoniae*; all complexes inhibited the adherent growth with MBECs ranging from 0.04 to 0.11 mM. Regarding the other tested strains, complexes (**3**) and (**4**) were generally more active as anti-biofilm species than complexes (**1**) and (**2**), with an MBEC of 0.14 mM for (**3**) and (**4**) against *B. subtilis* and of 0.07 mM against *C. albicans* (Table 7). The order of the anti-biofilm activity of the four complexes was similar to that obtained for the MIC: (**3**) > (**2**) > (**1**) > (**4**).

Overall, these data are consistent with an enhanced activity of Cu(II) species compared to those of Zn(II). For both metal ions, the obtained complexes with dmtp are more active than those with the bulky mptp in the case of planktonic and biofilm-embedded strains.

Notably, high activity was recorded for the obtained complexes against *K. pneumoniae*, *B. subtilis*, and *C. albicans* strains that had not previously been susceptible to [Cu(N-N)_2_(pmtp)](ClO_4_)_2_ and [Cu(N-N)(dmtp)_2_(OH_2_)](ClO_4_)_2_·dmtp complexes bearing tpds ligands [21,33]. Additionally, the activity of complex (**3**) against *P. aeruginosa* is enhanced in comparison with that of [Cu(N-N)(dmtp)_2_(OH_2_)](ClO_4_)_2_·dmtp complexes [33]. The same trend was observed concerning the anti-biofilm activity of the reported complexes. 

### 2.4. Complexes Interaction with ROS

The ability of the Cu(II) complexes to scavenge or trap reactive oxygen species (ROS) was investigated using EPR spectroscopy. A 10 mM solution of the samples was used in combination with KO_2_ as an O_2_^−^ source and H_2_O_2_ as an OH^−^ source, respectively.

**Figure 11:**
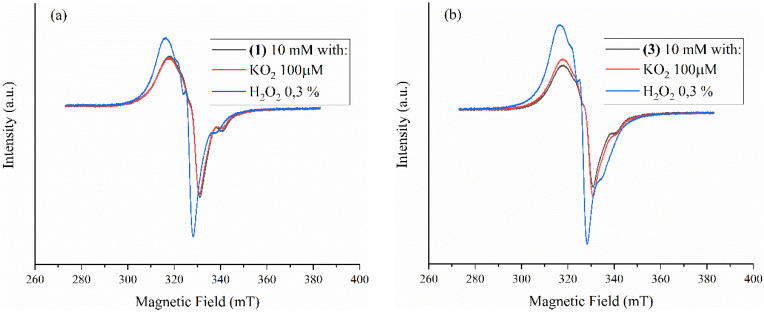
EPR spectra of complex (**1**) (**a**) and (**3**) (**b**) interacting with KO_2_ and H_2_O_2_ as O_2_^−^ and OH^−^ radical sources, respectively.

The EPR spectra are presented in Figure 11 and show the interaction between complexes (**1**) and (**3**) and the ROS species, as mentioned earlier. Both complexes show a meager capacity to trap or scavenge superoxide species; the intensity and shape of the EPR spectra do not change much. In contrast, both complexes show an excellent ability to trap OH^−^ radicals; the EPR spectra change considerably in intensity and shape.

### 2.5. Complexes Interaction with DNA

**Figure 12:**
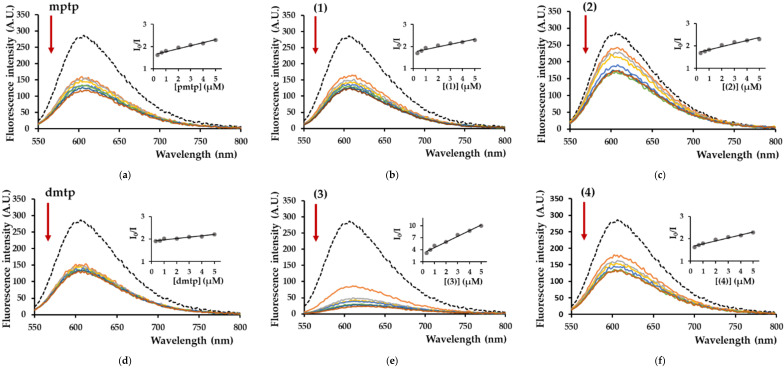
Fluorescence of λ-DNA/EB in the absence (black curves, dotted line) or in the presence of increasing concentrations of mptp (**a**); complex (**1**) (**b**); complex (**2**) (**c**); dmtp (**d**); complex (**3**) (**e**); complex (**4**) (**f**). Arrows indicate the quenching of the fluorescence and the increase in compound concentrations. Concentrations used: λ-DNA, 3 μM; EB, 1 μM; mptp, dmtp, (**1**)–(**4**), 1–10 μM. Inset: I_0_/I versus [quencher].

In most cases, the antiproliferative and antimicrobial traits of complexes are related to their DNA-binding and DNA-cleavage capacities, respectively. To check if our complexes bind to DNA, we determined their action upon the fluorescence of the DNA/EB (ethidium bromide) adduct. Ethidium bromide is one of the most utilized dyes for DNA detection as it causes intense fluorescence increase after intercalating into the DNA duplex. If a metal complex intercalates into the DNA, it leads to a decrease in the binding sites of DNA occupied by EB, and a reduction in the fluorescence intensity of the DNA/EB system can be observed [46]. Therefore, the complexes were added to λ-DNA (DNA of bacteriophage λ) that was pre-treated with EB. As shown in Figure 12, both mptp (Figure 12a) and dmtp (Figure 12d) could quench the fluorescence of the λ-DNA/EB adduct, indicating that the ligands themselves may bind to DNA. The fluorescence quenching caused by the free ligands was abrupt and did not vary much with concentration. The quenching caused by the exposure to complex (**1**) (Figure 12b) and (**4**) (Figure 12f) was similar to that caused by the corresponding ligands. Complex (**2**) caused weaker but more nuanced quenching, which varied with increasing concentration (Figure 12c). In contrast, complex (**3**) had the most substantial quenching ability (Figure 12e), indicating a stronger DNA interaction.

The emission intensity of EtBr bound to λ-DNA was quenched upon the incremental addition of both ligands and complexes and this quenching was linear in the concentration range of 0.3–5 μM (Figure 12, insets). This range of concentrations corresponds to a concentration ratio [quencher]/[DNA] of 0.1–1.66. Quenching data were analyzed according to the Stern–Volmer equation I_0_/I = 1 + K_SV_ [quencher] [47,48], where I_0_ and I are the emission intensity in the absence and in the presence of the quencher, respectively, K_SV_ is the Stern–Volmer constant, and [quencher] is the quencher concentration. K_SV_ was calculated from the slope of the plot of I_0_/I versus [quencher] (Figure 12a–f, insets). In the linear-fit plot, the K_SV_ determined for mptp, (**1**), (**2**), dmtp, (**3**), and (**4**) are 0.130, 0.115, 0.136, 0.056, 1.412, and 0.133 μM^−1^, respectively. These results indicate that complex (**3**) is the best quencher of λ-DNA-EB fluorescence, probably due to a stronger DNA-intercalating trait.

**Figure 13:**
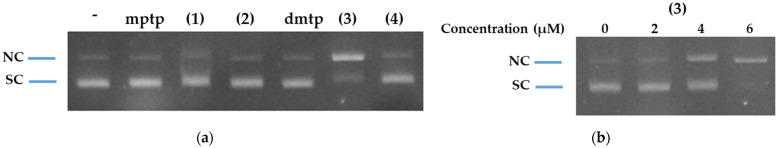
Gel electrophoresis image of pUC19 (100 ng/μL) after incubation for 1 h at 37 °C with the indicated compounds. (**a**) Lane 1: pUC19 alone; Lanes 2–7: pUC19 + 5 μM of indicated compounds. (**b**) Effect of (**3**) concentration on the relaxation of PUC19. SC, supercoiled; NC, nicked-circular.

The nuclease activity of complexes (**1**)–(**4**) was also tested. For this purpose, we used plasmid pUC19, which is a 2686 bp circular plasmid. Gel electrophoresis of purified pUC19 identified two main bands belonging to the supercoiled (SC) plasmid, which migrates faster, and the nicked-circular (NC) plasmid, which migrates slower (Figure 13). It was noted that only complex (**3**) caused DNA relaxation to the NC form (Figure 13a). This was expected, considering the more substantial fluorescence quenching that was noted for complex (**3**) (Figure 12e). We also tested the effect of the concentration of (**3**) on the DNA-cleavage activity; we noted that the NC band intensified at 4–6 μM, indicating some nuclease-like activity in this concentration range (Figure 13b).

The complexation with Zn(II) did not change the quenching traits of either mptp or dmtp (Figure 12a,c,d,f). Instead, the complexation with Cu(II) augmented the quenching ability of dmtp, which paralleled the onset of nuclease activity. The stronger interaction with DNA that was noticed for complex (**3**) may be the result of the less bulky methyl group presence, which is a characteristic that may increase its DNA-intercalating potential.

## 3. Materials and Methods

### 3.1. Reagents

The chemicals for synthesis of the complexes were purchased from Sigma-Aldrich (Darmstadt, Germany) (copper(II) chloride dihydrate (≥99.99% trace metals basis), zinc(II) chloride, 2,3-pentanedione (97%), 1-phenyl-1,3-butanedione (99%) and 3-amino-4H-1,2,4-triazole (96%)) and Merck (Darmstadt, Germany) (dibenzo-18-crown-6-ether, potassium superoxide) as reagent grade and were used as received, without further purification. The 5-methyl-7-phenyl-1,2,4-triazolo[1,5-*a*]pyrimidine (mptp) was obtained by [1 + 1] condensation of 3-amino-4H-1,2,4-triazole and 1-phenyl-1,3-butanedione while 5,7-dimethyl-1,2,4-triazolo[1,5-*a*]pyrimidine (dmtp) was synthesized by [1 + 1] condensation of 3-amino-4H-1,2,4-triazole and 2,3-pentanedione, as was reported in the literature [49].

### 3.2. Physical Measurements

A EuroEA elemental analyzer (Perkin Elmer, Waltham, MA, USA) was used for chemical analyses (C, N, and H). Fourier-transform-infrared-spectroscopy (FTIR) spectra were recorded in KBr pellets with a Tensor 37 spectrometer (Bruker, Billerica, MA, USA) in the 400–4000 cm^−1^ range. UV-Vis spectroscopy was performed in the solid state on a V 670 spectrophotometer (Jasco, Easton, MD, USA) with Spectralon as a standard in the 200–1500 nm range. The X-band Electron Paramagnetic Resonance (EPR) spectroscopy measurements were carried out with a continuous-wave X-Band Elexsys 580 EPR spectrometer (Bruker AXS GmbH, Karlsruhe, Germany) equipped with a Bruker X-SHQ 4119HS-W1 X-Band resonator. The measurement parameters for the X-Band measurements, if not otherwise mentioned, were set as follows: microwave frequency 9.88 GHz, microwave power 6.32 mW, modulation amplitude 0.2 mT, conversion time 40 ms, time constant 20.84 ms at 10 scans. ^1^H NMR and ^13^C NMR spectra were acquired by a Bruker Advance Ultrashield Plus 500 spectrometer (Bruker AXS GmbH, Karlsruhe, Germany) with a 500 MHz working frequency at 25 °C. Chemical shifts were measured in parts per million using the internal standard tetramethylsilane (TMS). Cyclic voltammetry experiments were carried out using a three-electrode configuration consisting of a glassy-carbon or platinum (disk, 3 mm diameter) as a working, Pt wire as the counter, and Ag/AgCl separated from the solution by a bridge filled with a 0.1 M Bu_4_NClO_4_ solution in DMSO as the reference electrode. All reported potentials are related to this reference electrode. The electrochemical measurements were performed using an Autolab PGSTAT 12, and the analysis was carried out with the GPES software. The working electrodes were polished with 0.3 μm alumina powder and then rinsed with distilled water before use. All the experiments were performed at room temperature for the studies carried out in an inert atmosphere before the electrochemical scan solutions were purged with Argon (99.9999%) for 10 min. Tetrabutylammonium perchlorate (Bu_4_NClO_4_) 0.1 M was used as a supporting electrolyte.

Crystallographic data were collected with an IPDS II diffractometer (STOE, Darmstadt, Germany) having a Mo-Kα (λ = 0.71073 Å) X-ray tube with a graphite monochromator. A crystal of suitable size was selected from the mother liquor and immersed in paratone oil, then mounted on the tip of a glass fiber and cemented using epoxy resin. Data collection: Stoe X-AREA. Cell refinement: Stoe X-AREA [50]. The structure was solved by direct methods and refined by full-matrix least-squares techniques based on F^2^. The non-H atoms were refined with anisotropic displacement parameters. Calculations were performed using the SHELX-2018 crystallographic software package [51]. A summary of the crystallographic data and the final refinement details are presented in Table 1. Crystallographic data (excluding structure factors) have been deposited with the Cambridge Crystallographic Data Centre with CCDC reference numbers 2124196 and 2124197. These data can be obtained free of charge via http://www.ccdc.cam.ac.uk/conts/retrieving.html (accessed on 23 December 2021), or from the Cambridge Crystallographic Data Centre, 12 Union Road, Cambridge CB2 1EZ, UK; Fax: +44-1223-336-033; or e-mail: deposit@ccdc.cam.ac.uk.

### 3.3. Synthesis and Characterization of the Complexes

[Cu(mptp)_2_Cl_2_] (**1**): To a solution containing copper(II) chloride dihydrate (0.085 g, 0.5 mmol) in 25 mL ethanol, a solution containing mptp (0.210 g, 1 mmol) and 0.1 mL DMSO in 30 mL ethanol was added. This mixture was magnetically stirred at 50 °C for 4 h and then filtered for green-precipitate removal. Blue crystals suitable for X-ray analysis were obtained after one month by slow evaporation of the obtained green solution. Analysis found: C, 43.83; H, 3.75; N, 21.68%; calculated for CuC_24_H_20_N_8_Cl_2_ (M_w_: 554.92 g mol^−1^): C, 51.95; H, 3.63; N, 20.19%, IR (KBr pellet, cm^−1^): υ(CH), 3137 w; υ_asym_(CH_3_), 2920 w; υ_sym_(CH_3_), 2870 w; υ(C=N)_trp_, 1615 m; υ(C=N)_pym_, 1543 vs; υ(Cu-N), 461 w, UV-Vis (solid, nm): π→π*, 285; CTLM, 360; d_x2−y2_ → d_xy_, 560; d_xz_ → d_xy_, 610; d_z2_ → d_xy_, 660.

[Zn(mptp)(DMSO)Cl_2_] (**2**): To a solution containing zinc(II) chloride (0.068 g, 0.5 mmol) in 25 mL ethanol, a solution containing mptp (0.210 g, 1 mmol) and 0.1 mL DMSO in 30 mL ethanol was added and the mixture was magnetically stirred at 50 °C for 4 h. The solution was filtered in order to remove the yellow, sparingly soluble species, and by slow evaporation of the yellow solution, crystals suitable for X-ray analysis were obtained after one month. Analysis found: C, 39.63; H, 3.75; N, 13.25; S, 7.46%; calculated for ZnC_14_H_16_N_4_SOCl_2_ (M_w_: 424.69 g mol^−1^): C, 39.59; H, 3.80; N, 13.19; S, 7.55%, IR (KBr pellet, cm^−1^): υ(CH), 3137 w, 3110 w, 3078 w; υ_asym_(CH_3_), 2916 w; υ_sym_(CH_3_), 2886 w; υ(C=N)_trp_, 1616 vs; υ(C=N)_pym_, 1544 vs; υ(S=O), 1026 m; υ(C-S), 722 w; υ(Cu-N), 457 w, UV-Vis (solid, nm): π→π*, 335; ^1^H NMR (500 Mz, DMSO d_6_, ppm): 2.54 (s, 3H, CH_3_-DMSO), 2.68 (s, 3H, CH_3_), 7.54 (s, H, CH_pyrim_), 7.64 (m, 3H, CH_ph_), 8.15 (d, 2H, CH_ph_), 8.61 (s, H, CH_triaz_); ^13^C NMR (500 Mz, DMSO d_6_, ppm): 16.94 (CH_3_), 24.62 (CH_3_-DMSO), 110.40 (C6), 128.65, 129.09, 129.50, 129.67, 131.53 (C phenyl ring), 146.17 (C7), 155.28 (C_bridge_, C2) and, 165.23 (C5).

[Cu_2_(dmtp)_4_Cl_4_]·2H_2_O (**3**): The compound was synthesized as previously published [22] with slight modifications. Analysis found: C, 37.51; H, 3.95; N, 25.05%; calculated for Cu_2_C_28_H_36_N_16_O_2_Cl_4_ (M_w_: 879.58 g mol^−1^): C, 37.47; H, 4.04; N, 24.97%, IR (KBr pellet, cm^−1^): υ_asym_(H_2_O), 3508 s; υ_sym_(H_2_O), 3445 s; υ(CH); υ_asym_(CH_3_), 2924 w; υ_sym_(CH_3_), 2856 w; υ(C=N)_trp_, 1626 vs; υ(C=N)_pym_, 1544 vs; υ(Cu-N), 488 w, UV-Vis (solid, nm): π→π*, 270; CTLM, 345; d_x2−y2_ → d_z2_, 750; d_xy_ → d_z2_, 910.

[Zn(dmtp)_2_Cl_2_] (**4**): The compound was synthesized as previously published [22,34] with slight modifications. Analysis found: C, 42.37; H, 3.75; N, 25.90%; calculated for ZnC_14_H_16_N_8_Cl_2_ (M_w_: 432.65 g mol^−1^): C, 38.87; H, 3.73; N, 25.90%, IR (KBr pellet, cm^−1^): υ(CH), 3120; υ_asym_(CH_3_), 2957 w; υ_sym_(CH_3_), 2921 w; υ(C=N)_trp_, 1630 vs; υ(C=N)_pym_, 1543 vs; υ(Cu-N), 487 w, UV-Vis (solid, nm): π→π*, 270; ^1^H NMR (500 Mz, DMSO d_6_, ppm): 2.58 (s, 3H, CH_3_), 2.73 (s, 3H, CH_3_), 7.18 (s, H, CH_pyrim_), 8.56 (s, H, CH_triaz_); ^13^C NMR (500 Mz, DMSO d_6_, ppm): 16.45 (CH_3_), 24.47 (CH_3_), 110.90 (C6), 147.04 (C7), 155.06 (C_bridge_, C2) and, 164.54 (C5).

### 3.4. Biological Characterization of Compounds

#### 3.4.1. Screening of the Antimicrobial Properties

The antimicrobial assays were carried out using two Gram-negative (*K. pneumoniae* ATCC 134202, *P. aeruginosa* ATCC 27853) and Gram-positive (*B. subtilis* ATCC 6633) reference, as well as the *C. albicans* 22 strains. The antimicrobial activity of the complexes *versus* the ligands and metal salts against planktonic microbial cells was assessed using the microdilution assay, allowing the MIC determination [52,53,54]. The anti-biofilm activity was evaluated using the crystal-violet microtiter to determine the MBEC [30,31,32,33]. All experiments were performed in triplicate. In both assays, serial binary concentrations ranging from 4.76 to 0.04 mM were used.

#### 3.4.2. In Vitro Cytotoxicity Assay

##### Cell Culture Conditions

Mouse melanoma cells (B16—ATCC CRL-6475, USA) were grown in DMEM (Dulbecco’s Modified Eagle Medium) supplemented with 2 mM L-Glutamine, 10% fetal calf serum (FCS), 100 units/mL of penicillin, and 100 µg/mL of streptomycin at 37 °C in a humidified incubator under an atmosphere containing 5% CO_2_. All cell-cultivation media and reagents were purchased from Biochrom AG (Berlin, Germany) and Sigma-Aldrich (Darmstadt, Germany).

##### Cell Viability Assay

Cell viability was evaluated using 3-(4,5-dimethylthiazol-2-yl)-2,5-diphenyltetrazolium bromide (MTT) assay as previously described [21,33]. The influence of the tested complexes was evaluated after 24 h of contact with concentrations varying from 1 to 75 μM. The negative control was represented by cells cultivated only in medium, without the investigated compounds. The data were corrected for the background, and the percentage of viable cells was obtained using the equation:

Cell viability = [(A_570_ of treated cells)/(A_570_ of untreated cells)] × 100%

The data processing was performed using Origin 8.1 (Microcal Inc. Los Angeles, CA, USA).

##### Phalloidin Staining and Cell Imaging

According to the manufacturer protocol, the cytoskeleton actin filaments of B16 cells were stained with phalloidin-FITC (Sigma-Aldrich, USA). Briefly, cells were washed with PBS (5 min, 3 times), fixed for 5 min with 3% paraformaldehyde, washed three times with PBS, permeabilized with 0.1% Triton X-100 in PBS for 15 min, washed three times with PBS, stained with 20 μg mL^−1^ phalloidin-FITC at room temperature for 1 h and rewashed three times with PBS. The cell nucleus was stained with 8 μM of Hoechst 33,342 solution for 10 min, washed three times with PBS, and finally, mounted and sealed on glass slides with FluorSave™ Reagent (Merck Millipore, Germany). The fluorescence images were acquired using an Olympus DP74 (Olympus, Germany), mounted on an epifluorescence microscope, Olympus BX-51 (Olympus, Germany), equipped with a 40× objective and an appropriate DAPI/Hoechst filter cube and GFP/FITC filter cube.

#### 3.4.3. Interaction with Biological Species

##### Superoxide Scavenging Ability

The superoxide-scavenging ability of the complexes was tested using the KO_2_ compound as a superoxide source combined with EPR spectroscopy. To carry out the experiments, a 10 µM DMSO solution of the complexes was mixed with different concentrations of KO_2_, and the EPR signal-intensity changes were monitored. The KO_2_ was dissolved by complexation with dibenzo-18-crown-6-ether.

##### Fluorescence of λ-DNA/Ethidium Bromide Adduct

To record the DNA/EB fluorescence spectra, we used the total DNA isolated from bacteriophage λ (λ-DNA, Promega, Madison, WI, USA). The binding of compounds to λ-DNA was assayed by monitoring the quenching of fluorescence emitted by λ-DNA/EB adduct (λexcit = 510). The λ-DNA solution (3 μM in Tris-HCl/NaCl buffer solution) was first added to the EB solution prepared in the same buffer, then increasing concentrations of compounds (**1**)–(**4**) were added to λ-DNA/EB [55,56]. The fluorescence spectra were recorded in the range of 550–800 nm using a Thermo Fischer Scientific Varioskan Flash spectral scanning multimode reader (Vantaa, Vantaa, Finland). The spectra were recorded in suitable plates using 5 nm excitation and emission slits for all measurements.

##### Nuclease-Like Activity Assay

The nuclease activity of the compounds was determined using plasmid pUC19 DNA. The plasmid was amplified in *Escherichia coli* by transforming One Shot^®^ TOP10 chemically competent *E. coli* (Invitrogen, Thermo Fisher Scientific, Waltham, MA, USA). The plasmid was isolated from positive colonies using a PureLink™ HiPure Plasmid Miniprep Kit (Invitrogen, Thermo Fisher Scientific). The plasmid (100 ng/sample) was exposed to 5 µM compounds. The DNA-cleavage experiments for the complexes were performed in a 9/1 (*v*/*v*) ratio of 50 mM Tris-HCl buffer, pH 8, and DMSO. The samples were incubated for 1 h at 37 °C before a bromophenol blue/xylene cyanol-based loading dye (Roth, Germany) was added to the samples. The samples were loaded on a 1% (*w*/*v*) agarose gel containing 1 μg/mL EtBr in 1 × TAE (Tris–acetic acid–EDTA) buffer. Electrophoresis was performed at 100 V for 45 min in 1 × TAE buffer. The images of the fluorescent ethidium bromide-stained gels were captured using a gel-documentation system (Doc-Print II, VilberLourmat, France). The cleavage experiments were performed six times with similar results. One representative gel is shown.

## 4. Conclusions

A series of complexes with 1,2,4-triazolo[1,5-a]pyrimidine derivatives was studied as potential biologically active species. Complexes (**1**) and (**2**) were fully characterized by single X-ray diffraction, a method that evidenced a square-planar stereochemistry in the Cu(II) complex and tetrahedral in the Zn(II) compound, and a supramolecular network generated by π–π or C-H∙∙∙π interactions. The EPR spectra are consistent with X-ray diffraction’s stereochemistries evidenced for the Cu(II) ions. The complexes’ stability in DMSO solution at times corresponding to biological assays was proved through EPR and NMR spectra. The complexes exhibited a moderate antiproliferative effect against the B16 tumor cells since the viability was reduced by 80% at concentrations higher than 75 µM. The results of antimicrobial activity demonstrate that the tested complexes exhibit significant antibacterial and antifungal activity at very low concentrations ranging from 0.04 to 0.45 mM, with complex (**3**) harboring the best antimicrobial and antibiofilm properties. Moreover, all complexes are very active against the *K. pneumoniae* strain, known as one of the most fearful etiological agents of opportunistic infections in cancer patients. The potential mechanism of action could be the nuclease-like activity of complexes, which is also supported by the complexes’ ability to generate ROS. These data recommend complex (**3**) for further study to develop new materials with high antimicrobial activity.

## Figures and Tables

**Table 1:** Crystal data and structure refinement for compounds (**1**) and (**2**).

Empirical Formula	C_12_H_11_ClCu_0_._5_N_4_ (1)	C_14_H_17_Cl_2_N_4_OSZn (2)
Formula weight	278.48	425.64
Temperature/K	293(2)	293(2)
Crystal system	*monoclinic*	*monoclinic*
Space group	*P*2_1_/n	*P*2_1_/n
a/Å	9.1524(12)	13.1535(8)
b/Å	14.0262(14)	7.6713(4)
c/Å	10.3212(13)	18.0452(8)
α/°	90	90
β/°	115.602(15)	93.025(5)
γ/°	90	90
Volume/Å^3^	1194.9(3)	1818.31(16)
Z	4	4
ρ_calc_g/cm^3^	1.542	1.555
μ/mm^−1^	1.166	1.767
F(000)	570.0	868.0
Radiation	(λ = 0.71073)	(λ = 0.71073)
2Θ range for data collection/°	5.252 to 60.824	3.932 to 61.588
Reflections collected	4901	9717
Independent reflections	2570 [R_int_ = 0.0254, R_sigma_ = 0.0368]	4416 [Rint = 0.0312, Rsigma = 0.0451]
Data/restraints/parameters	2570/0/161	4416/0/211
Goodness-of-fit on F^2^	1.074	1.071
Final R indexes [I ≥ 2σ (I)]	R_1_ = 0.0442, wR_2_ = 0.1356	R_1_ = 0.0386, wR_2_ = 0.0897
Final R indexes [all data]	R_1_ = 0.0513, wR_2_ = 0.1418	R_1_ = 0.0643, wR_2_ = 0.0975
Largest diff. peak/hole/e Å^−3^	0.66/−0.73	0.31/−0.52

**Table 2:** Selected bond lengths (Å) and bond angles (°) for complexes.

(1)			(2)		
Cu1	Cl1 ^1^	2.2583(8)	Zn1	Cl2	2.2133(8)
Cu1	Cl1	2.2583(8)	Zn1	Cl1	2.2234(9)
Cu1	N1	1.986(2)	Zn1	N1	2.033(2)
Cu1	N1 ^1^	1.986(2)	Zn1	O1	1.9921(18)
N3	N2	1.370(3)	S1	C4	1.769(4)
N3	C2	1.378(3)	S1	C6	1.767(3)
N3	C7	1.372(3)	S1	O1	1.5224(19)
C3	C2	1.339(4)	N1	C12	1.352(3)
C3	C4	1.330(4)	N1	C14	1.351(3)
N2	C1	1.314(4)	N2	N3	1.366(3)
N1	C2	1.341(3)	N2	C14	1.303(3)

Symmetry transformations used to generate equivalent atoms: ^1^ 1-*x*, 1-*y*, -*z.*

**Table 3:** The M-N and M-Cl (M: Cu, Zn) bond lengths (Å) for complexes (**1**)–(**4**).

Compound	M-N_tpds_	M-Cl	Ref.
(**1**)	1.986(2)	2.2583(8)	This paper
(**2**)	2.033(2)	2.2133(8) 2.2234(9)	This paper
(**3**)	1.9997(15) 2.005(15)	2.2831(6) 2.3993(6)	[22]
(**4**)	2.020(2) 2.058(2)	2.197(1) 2.2499 (8)	[34]

**Table 4:** Cyclic-voltammetry data for complexes (**1**), (**3**), [Cu(DMSO)_6_]Cl_2_ and, ligands mptp and dmtp.

Compound	E_pa1_ (V)	E_pc1_ (V)	E_pc2_ (V)	Ep_a2_ (V)	E_1/2_ (V) * (Cu(III)/Cu(II))
[Cu(DMSO)_6_]Cl_2_	+0.421	+0.200	−0.766	−0.061	+0.311
mptp	-	-	−0.705	−0.796	
(**1**)	+0.482	+0.220	-	-	+0.351
dmtp	-	-	−0.876	−0.705	
(**3**)	+0.583	+0.200	−0.846	-	+0.391

All potentials are measured in V vs. Ag/AgCl/(0.1 Bu_4_NClO_4_ in DMSO); * Half-wave potentials were calculated for Cu(III)/Cu(II) couple by using E_1/2_ = (E_pa1_ + E_pc1_)/2.

**Table 5:** Cyclic voltammetry data for complexes (**2**), (**4**), [Zn(DMSO)_4_]Cl_2_ and ligands mptp and dmtp.

Compound	E_pc1_ (V)	E_pc2_ (V)	E_pa1_ (V)	E_pa2_ (V)
[Zn(DMSO)_4_]Cl_2_	−0.654	-	−1.037	-
mptp	-	−1.591	1.501	-
(**2**)	−0.654	−1.531	-	-
dmtp	−0.725	−2.014	−1.924	−0.342
(**4**)	−0.644	−1.742	-	-

All potentials are measured in V vs. Ag/AgCl/(0.1 Bu_4_NClO_4_ in DMSO).

**Table 6:** The MIC values (mM) for ligand, metal chlorides, and complexes.

Bacterial Strain	mptp	(1)	(2)	CuCl_2_·2H_2_O	ZnCl_2_	dmtp	(3)	(4)
*K. pneumoniae* 134202	2.38	0.23	0.15	5.87	7.35	3.42	0.14	0.07
*P. aeruginosa* 27853	4.76	0.45	-	5.87	7.35	6.85	0.28	1.12
*B. subtilis* 6633	2.38	0.23	0.29	5.87	7.35	3.42	0.14	0.14
*C. albicans* 22	-	-	0.29	5.87	7.35	3.42	0.07	0.29
Average MIC value	3.173	0.303	0.243	5.870	7.350	4.277	0.158	0.405

**Table 7:** The compounds influence on biofilm formation (MBEC value, mM).

Bacterial Strain	mptp	(1)	(2)	CuCl_2_·2H_2_O	ZnCl_2_	dmtp	(3)	(4)
*K. pneumoniae* 134202	2.38	0.11	0.04	>5.87	7.35	3.42	0.07	0.07
*P. aeruginosa* 27853	4.76	0.45	-	0.36	0.45	6.85	0.28	1.12
*B. subtilis* 6633	2.38	0.23	0.29	5.87	7.35	3.42	0.14	0.14
*C. albicans* 22	-	-	0.29	5.87	7.35	3.42	0.07	0.29
Average MBEC value	3.173	0.263	0.207	4.492	5.625	4.277	0.140	0.405

## Data Availability

Not applicable.

## Supplementary Materials

The following supporting information can be downloaded online. Figure S1: UV-Vis spectra of complexes (**1**) (dark blue), (**2**) (yellow) and mptp (black), Figure S2: UV-Vis spectra of complexes (**3**) (green), (**4**) (yellow) and dmtp (black), Figure S3: EPR spectra of complexes (**1**) and (**3**) in 1 and 10 mM DMSO solution, Figure S4: ^1^H NMR spectra of fresh prepared complex (**2**) (green) and after 72 h (red)., Figure S5. ^13^C NMR spectrum of complex (**2**), Figure S6: ^1^H NMR spectra of fresh prepared complex (**4**) (green) and after 72 h (red), Figure S6. ^13^C NMR spectrum of complex (**4**), Table S1: Selected geometric parameters-angles (°) in compounds (**1**) and (**2**).

## Sample Availability

Samples of the compounds (**1**)–(**4**) are available from the authors.
