# Supplementary material for: Insights into Structure and Biological Activity of Copper(II) and Zinc(II) Complexes with Triazolopyrimidine Ligands
Source: Molecules. 2022 Jan 24;27(3):765. doi: 10.3390/molecules27030765 (PMC8838430; doi:10.3390/molecules27030765)
Supplement: Supplementary file 1 [file molecules-27-00765-s001.zip › molecules-1548407-supplementary.pdf]

molecules

Insights into structure and biological activity of copper(II) and zinc(II) complexes with triazolopyrimidine ligands

Aura Argăseală [1], Cătălin Maxim [1], Mihaela Badea [1], Larisa Ioniță [2], Mariana Carmen Chifiriuc [3,4], Arpad Mihai Rostas [5], Mihaela Bacalum [6], Mina Răileanu [6,7], Lavinia L. Ruţă [8], Ileana C. Farcaşanu [8], Emilia Elena Iorgulescu [9]*, and Rodica Olar [1]*

[1] University of Bucharest, Faculty of Chemistry, Department of Inorganic Chemistry, 90-92 Panduri Str., 050663 Bucharest, Romania; aura.argaseala@s.unibuc.ro (A.A.); catalin.maxim@chimie.unibuc.ro (C.M.); mihaela.badea@chimie.unibuc.ro (M.B.); rodica.olar@chimie.unibuc.ro (R.O.)
[2] Doping Control Laboratory, 37-39 Basarabia Blvd., 022103 Bucharest, Romania, l.ionita@lcd.gov.ro (L.I.)
[3] University of Bucharest, Faculty of Biology, Department of Microbiology, 1-3 Aleea Portocalelor Str., 060101 Bucharest, Romania; carmen.chifiriuc@bio.unibuc.ro (M.C.C.)
[4] Academy of Romanian Scientists, Bucharest, Romania; carmen.chifiriuc@bio.unibuc.ro (M.C.C.)
[5] National Institute of Materials Physics, Laboratory of Atomic Structures and Defects in Advanced Materials, 405A Atomiştilor Str., Măgurele-Ilfov, 077125, Romania; arpad.rostas@infim.ro (A.M.R.)
[6] Horia Hulubei National Institute for Physics and Nuclear Engineering, Department of Life and Environmental Physics, 30 Reactorului Str., Măgurele-Ilfov, 077125, Romania; bmihaela@nipne.ro (M.B.); mina.raileanu@nipne.ro (M.R.)
[7] University of Bucharest, Faculty of Physics, Department of Electricity, Solid State and Biophysics, 405A Atomiştilor Str., Măgurele-Ilfov, 077125, Romania
[8] University of Bucharest, Faculty of Chemistry, Department of Organic Chemistry, Biochemistry and Catalysis, 90-92 Panduri Str., 050663 Bucharest, Romania; lavinia.ruta@chimie.unibuc.ro (L.L.R); ileana.farcasanu@chimie.unibuc.ro (I.C.F)
[9] University of Bucharest, Faculty of Chemistry, Department of Analytical Chemistry, 90-92 Panduri Str., 050663 Bucharest, Romania; emilia-elena.iorgulescu@chimie.unibuc.ro (E.E.I.)

* Correspondence: emilia-elena.iorgulescu@chimie.unibuc.ro (E.E.I.), odica.olar@chimie.unibuc.ro (R.O.)

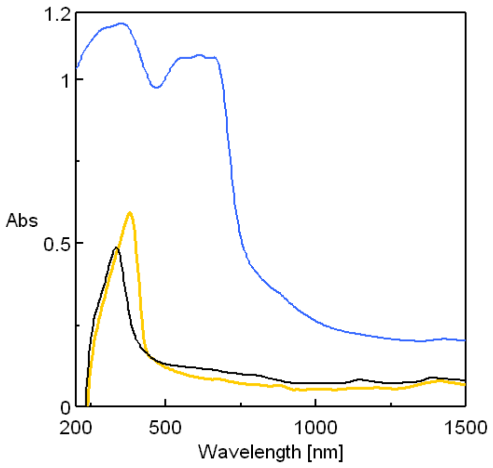

Figure S1. UV-Vis spectra of complexes (**1**) (dark blue), (**2**) (yellow) and pmtp (black).

molecules

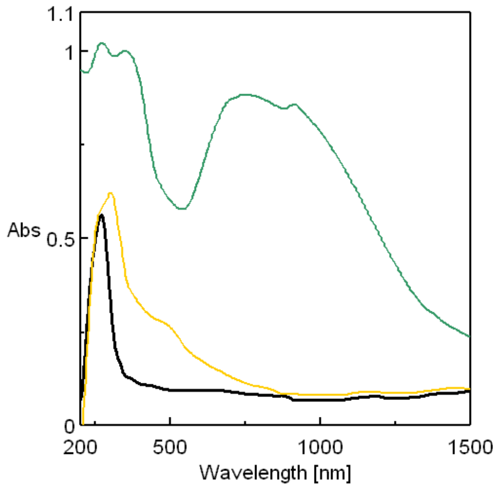

Figure S2. UV-Vis spectra of complexes (**3**) (green), (**4**) (yellow) and dmtp (black).

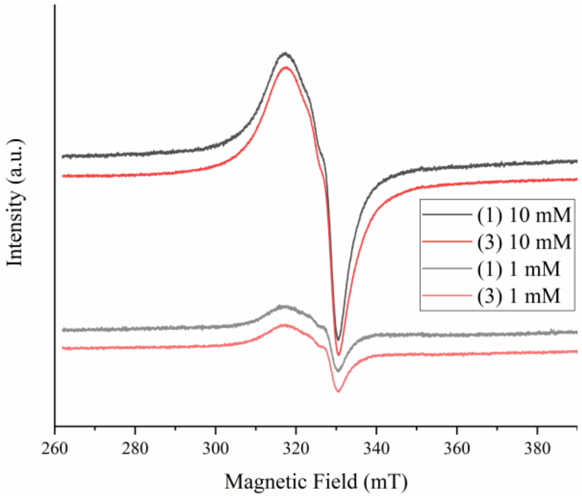

Figure S3. EPR spectra of complexes (**1**) and (**3**) in 1 and 10 mM DMSO solution.

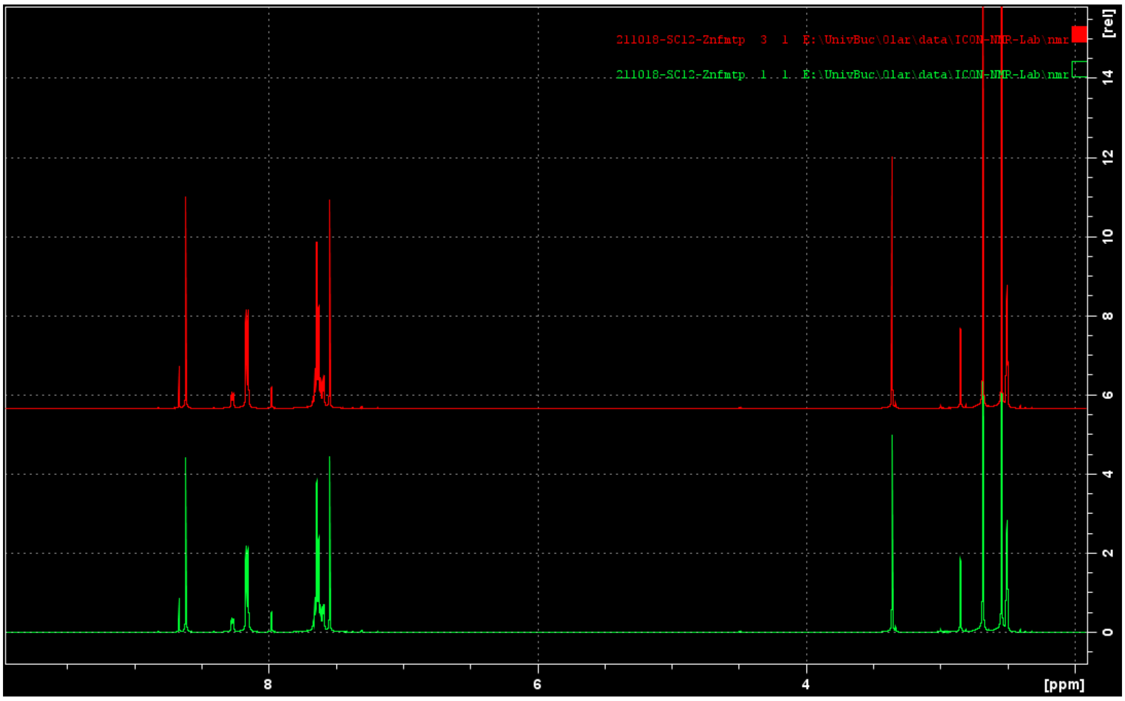

Figure S4. ^{1}H NMR spectra of fresh prepared complex (**2**) (green) and after 72 h (red).

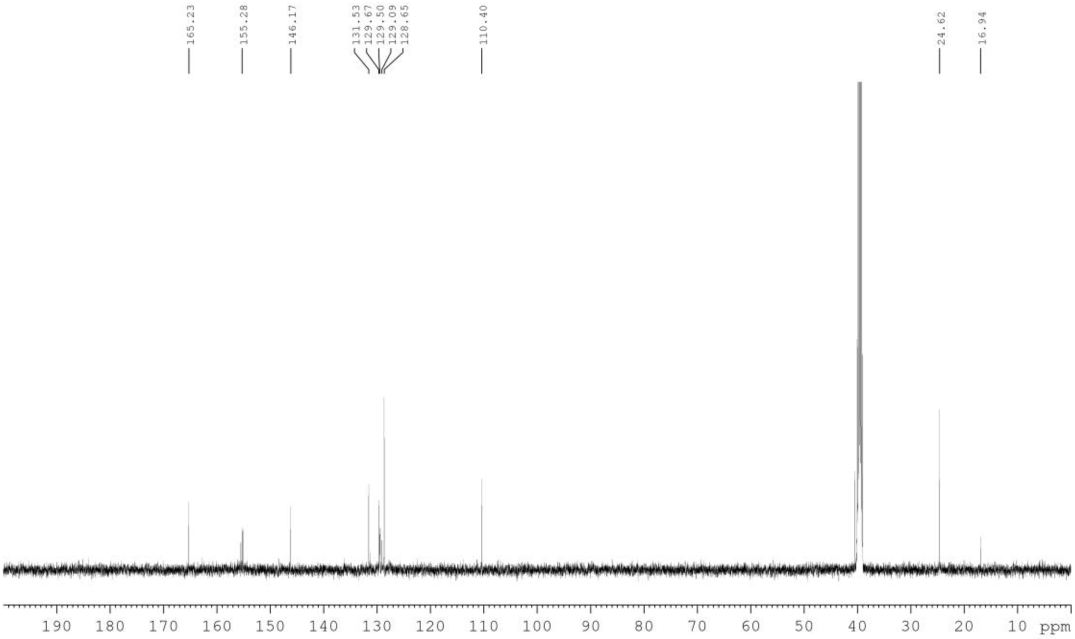

Figure S5. ^{13}C NMR spectrum of complex (**2**).

molecules MDPI

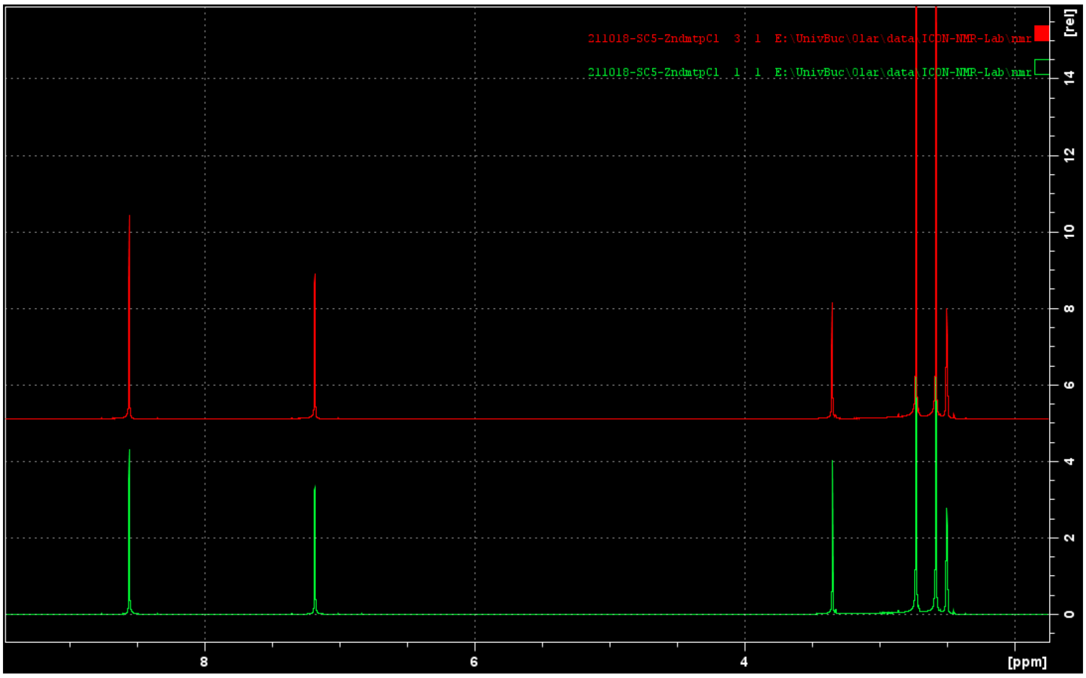

Figure S6. ^{1}H NMR spectra of fresh prepared complex (**4**) (green) and after 72 h (red).

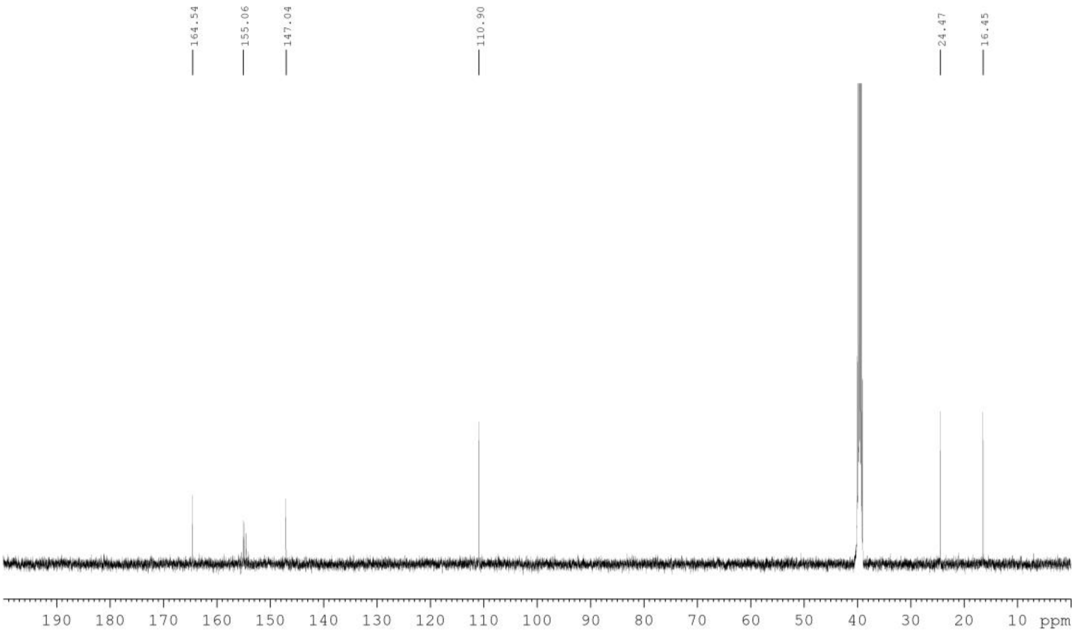

Figure S7. ^{13}C NMR spectrum of complex (**4**).

molecules MDPI

Table S1. Selected geometric parameters-angles (°) in compounds (**1**) and (**2**).

(1)				(2)			
Cl1	Cu1	Cl1[1]	180.0	O1	Zn1	Cl1	107.95(7)
N1	Cu1	Cl1	88.38(8)	O1	Zn1	N1	106.65(8)
N1[1]	Cu1	Cl1[1]	88.38(8)	C6	S1	C4	99.30(19)
N1	Cu1	Cl1[1]	91.62(8)	O1	S1	C4	103.79(16)
N1[1]	Cu1	Cl1	91.62(8)	O1	S1	C6	104.19(15)
N1[1]	Cu1	N1	180.0	C12	N1	Zn1	129.18(18)
N2	N3	C2	110.5(2)	C14	N1	Zn1	124.95(17)
N2	N3	C7	127.5(2)	C14	N1	C12	103.9(2)
C7	N3	C2	122.0(2)	C14	N2	N3	103.0(2)
C4	C3	C2	115.8(2)	N1	C12	N3	107.5(2)
C1	N2	N3	102.2(2)	N4	C12	N1	128.0(2)
C2	N1	Cu1	132.41(19)	N4	C12	N3	124.5(2)
C2	N1	C1	104.4(2)	C10	N4	C12	115.4(2)
C1	N1	Cu1	122.97(19)	N2	N3	C12	110.07(19)
C3	C2	N3	123.5(2)	N2	N3	C8	128.2(2)

Symmetry transformations used to generate equivalent atoms: [1]1-*x*, 1-*y*, -*z*
